# Utilization of Robotic Exoskeleton for Overground Walking in Acute and Chronic Stroke

**DOI:** 10.3389/fnbot.2021.689363

**Published:** 2021-09-01

**Authors:** Karen J. Nolan, Kiran K. Karunakaran, Pamela Roberts, Candy Tefertiller, Amber M. Walter, Jun Zhang, Donald Leslie, Arun Jayaraman, Gerard E. Francisco

**Affiliations:** ^1^Kessler Foundation, Center for Mobility and Engineering Research, West Orange, NJ, United States; ^2^Rutgers—New Jersey Medical School, Department of Physical Medicine and Rehabilitation, Newark, NJ, United States; ^3^Cedars-Sinai Medical Center, Department of Physical Medicine and Rehabilitation, Los Angeles, CA, United States; ^4^Craig Hospital, Department of Physical Therapy, Englewood, CO, United States; ^5^Sheltering Arms Physical Rehabilitation Centers, Mechanicsville, VA, United States; ^6^St. Charles Hospital, Port Jefferson, NY, United States; ^7^Shepherd Center, Atlanta, GA, United States; ^8^Shirley Ryan AbilityLab, Max Nader Center for Rehabilitation Technologies and Outcomes Research, Chicago, IL, United States; ^9^Department of Physical Medicine and Rehabilitation, Northwestern University, Chicago, IL, United States; ^10^University of Texas at Houston McGovern Medical School, Houston, TX, United States; ^11^TIRR Memorial Hermann, Houston, TX, United States

**Keywords:** gait, stroke, rehabilitation, wearable robotics, hemiplegia, exoskeleton, walking speed

## Abstract

Stroke commonly results in gait deficits which impacts functional ambulation and quality of life. Robotic exoskeletons (RE) for overground walking are devices that are programmable to provide high dose and movement-impairment specific assistance thus offering new rehabilitation possibilities for recovery progression in individuals post stroke. The purpose of this investigation is to present preliminary utilization data in individuals with acute and chronic stroke after walking overground with an RE. Secondary analysis on a subset of individuals is presented to understand the mechanistic changes due to RE overground walking. Thirty-eight participants with hemiplegia secondary to stroke were enrolled in a clinical trial conducted at eight rehabilitation centers. Data is presented for four sessions of overground walking in the RE over the course of 2 weeks. Participants continued their standard of care if they had any ongoing therapy at the time of study enrollment. Gait speed during the 10 Meter Walk Test, Gait deviations and the Functional Ambulation Category (FAC) data were collected before (baseline) and after (follow-up) the RE walking sessions. Walking speed significantly increased between baseline and follow-up for participants in the chronic (*p* <0.01) and acute (*p* < 0.05) stage of stroke recovery. FAC level significantly improved (*p* < 0.05) and there were significantly fewer (*p* < 0.05) gait deviations observed for participants in the acute stages of stroke recovery between baseline and follow-up. Secondary analysis on a subset of eight participants indicated that after four sessions of overground walking with the RE, the participants significantly improved their spatial symmetry. The walk time, step count and ratio of walk time to up time increased from first session to the last session for participants in the chronic and acute stages of stroke. The RE was effectively utilized for overground walking for individuals with acute and chronic stroke with varying severity levels. The results demonstrated an increase in walking speed, improvement in FAC and a decrease in gait deviations (from baseline to follow-up) after four sessions of overground walking in the RE for participants. In addition, preliminary data indicated that spatial symmetry and step length also improved after utilization of an RE for overground walking.

## Introduction

Stroke is a leading cause of severe disability in adults, affecting ~15 million people each year worldwide (Association, 2013). Individuals with stroke often present with gait and balance deficits, leading to activity limitations and participation restrictions (Wade and Hewer, 1987; Friedman, 1990). Regaining independent ambulation is a priority among acute and chronic stroke patients.

Current post stroke rehabilitation strategies can be effective but individuals are often left with residual gait deviations, resulting in compensatory mechanisms such as hip circumduction, toe walking, hip hiking, among others for ambulation. These gait deviations and pathological compensations often result in inefficient gait and reduced speed, which can negatively affect community ambulation.

Post stroke rehabilitation is based on the theory that repeated task specific mass practice will lead to recovery of ambulatory function (Partridge et al., 2000; Cooke et al., 2010). In addition, the closer the practiced task (reciprocal stepping) is to the functional goal (healthy walking) better the learning transfer and ultimately recovery. Therefore some of the critical parameters for improving mobility post stroke are activity-specific, mass practice that is progressively more challenging (Langhorne et al., 2009).

Stroke research has shown that improvements in functional outcome measures, such as increased walking speed, are strong predictors of independent community ambulation. Individuals with walking speeds of 0.4–0.8 m/s are classified as limited community ambulators, <0.4 m/s are classified as household ambulators, and >0.8 m/s are classified as community ambulators on all surfaces (Perry et al., 1995). The walking speed of individuals during the chronic stages of stroke recovery has been reported to be between the range of 0.3 and 0.8 m/s (Hill et al., 1997; Duncan et al., 1998; Eng et al., 2002; Green et al., 2002), classifying them as either household ambulators or limited community ambulators (Hill et al., 1997; Duncan et al., 1998; Eng et al., 2002; Green et al., 2002).

Another clinical assessment scale, the Functional Ambulation Category (FAC), distinguishes six levels of walking ability based on the amount of physical support required during ambulation. The FAC is a quick visual measurement of walking, and it correlates with walking speed and step length (Holden et al., 1984, 1986).

Though walking speed and FAC could indicate deficits and improvements in functional ambulation, they do not provide information about the underlying impairments in mechanisms or inter-limb coordination during recovery. Observational gait deviations, temporal and spatial characteristics have been previously studied to understand and quantify the underlying mechanisms associated with pathological compensations. Research has shown that temporal and spatial asymmetry are significant predictors of hemiparetic walking performance such as walking speed and falls in adults (Roth et al., 1997; Patterson et al., 2010). Individuals with stroke often present with higher asymmetries between their limbs, leading to ambulatory deficits (Patterson et al., 2010). Quantifying the change in temporal and spatial characteristics would help us further understand the effect of using RE on functional/clinical outcomes such as falls (Hausdorff et al., 2001). Understanding these characteristic changes will help further improve the design of rehabilitation interventions for individuals with deficits (Wall and Turnbull, 1986).

Commercial wearable robotic exoskeletons (RE) for overground walking offer new rehabilitation possibilities by providing task specific, high repetitive practice for individuals with acute and chronic stroke (Molteni et al., 2021). In addition, most of the REs can also provide stability and balance to keep the users in an upright position even in users with severe balance and gait deficits who are unable to maintain upright posture. REs are anthropomorphic mobile electromechanical devices usually powered bilaterally by two electric motors at the knee and hip joints (Dollar and Herr, 2008). The motorized movement trajectories at the hip and knee reduces the need for manual range of motion guidance by a physical therapist during motor rehabilitation. This allows physical therapists to focus on training cues and feedback to drive gait quality. During overground walking, the RE can assist gait initiation and limb advancement with complete or partial assistance and the support structure provides stability and balance. For individuals with moderate to severe impairments the RE provides reciprocal motor assistance including limb coordination (symmetry), and proprioceptive input during limb loading (Rojek et al., 2020). These mechanisms are critical elements for the recovery of independent ambulation post stroke.

Robotic exoskeletons (REs) have been used for rehabilitation during the chronic and subacute stages of stroke (Louie and Eng, 2016; Høyer et al., 2020; Molteni et al., 2021). As new rehabilitation robotic technology emerges for acute and chronic stroke rehabilitation, it is important to investigate the feasibility and safety of the device in order to expand their utility in clinical rehabilitation. Therefore, the purpose of this multicenter investigation is to present preliminary utilization data after walking overground in a robotic exoskeleton (RE) for acute and chronic post stroke patients. Secondary analysis is presented to begin to understand the functional changes and mechanistic changes in temporal and spatial characteristics and symmetry due to RE overground walking.

## Methods

### Participants

Individuals diagnosed with unilateral post stroke hemiplegia (*n* = 42) were enrolled into a prospective clinical trial across eight rehabilitation centers. Participants were evaluated by a licensed physical therapist and medical clearance for full weight bearing and locomotor training was obtained from a physician prior to enrollment. Participants were screened based on the following inclusion criteria: (1) over 18 years of age; (2) height between 5′1″ and 6′3″ and weight equal to or <250 lbs.; (3) able to walk 14 m with assistance from not more than 1–2 persons; (4) manual muscle test (MMT) score of 4/5 in at least one upper extremity; (5) have no other known brain abnormalities or neurological diseases or disorders; (6) not diagnosed or treated for more than one stroke; (7) have a passive joint range of motion (PROM) within normal functional limits for safe ambulation; (8) intact skin where the device is in contact with the participant; (9) Modified Ashworth Scale 3 or less in the involved (or paretic) lower extremity; (10) not have any complicating physical or mental conditions as determined by a physician that would prevent participation; (11) able to follow directions and communicate basic needs; (12) not have a colostomy bag; or (13) not have uncontrolled hypo/hypertension. The Institutional Review Board at each participating institution approved the investigation, and all participants consented prior to participation in the investigation.

**Figure 1:**
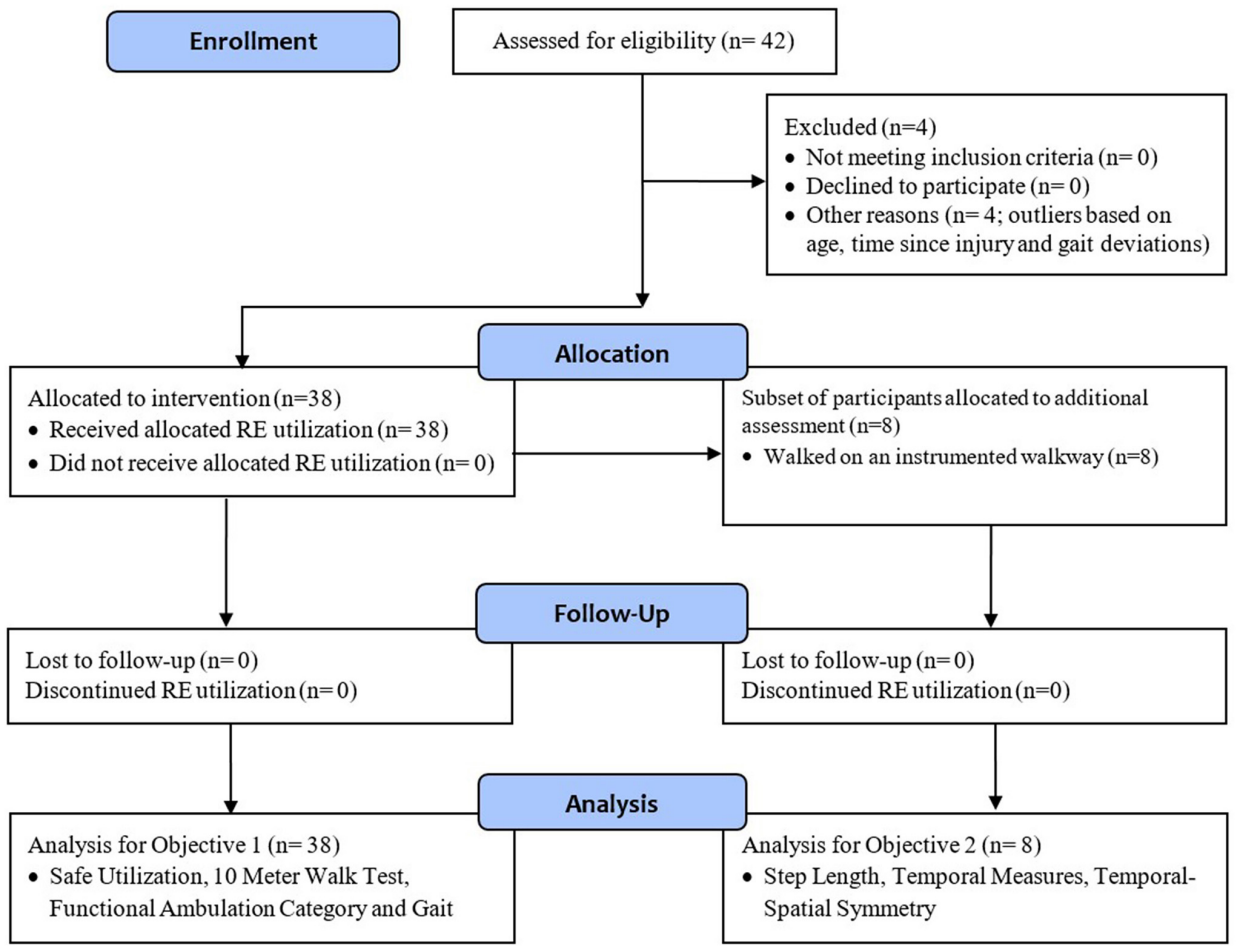
Consort diagram.

Four participants were removed as outliers from further analysis based on age, time since injury, and gait deviations at baseline. All outliers were identified as extreme outliers based on interquartile range in IBM SPSS (Figure 1). Data from 38 participants across eight rehabilitation centers were used for all further analysis, eight participants were acute (<6 months post injury), and 30 participants were chronic (>6 months post injury). Demographic information including age, height, weight, gender, affected side, and time since stroke were collected at baseline (Table 1). The Fugl-Meyer Assessment of Motor Function after Stroke (FMA) (Sullivan et al., 2011) was collected at baseline as a measure of lower extremity motor and sensory impairment. Participants categorized as acute and chronic had a range of 7–29 points and 15–34 points respectively on the Fugl-Meyer lower extremity assessment scale. The participants had varied impairment post stroke ranging from mild to very severe. The Face, Legs, Activity, Cry. Consolability (FLACC) is a behavioral pain assessment scale used for self-report of pain. The FLACC score was collected at the beginning of the session to monitor pain and the values for baseline and follow-up are presented in Table 1.

**Table 1:** Participant demographics, presented as mean ± standard deviation.

**Demographics**	**Acute stroke participants** ***n* = 8**	**Chronic stroke participants** ***n* = 30**
Age (years)	61.38 ± 14.51	60.33 ± 10.61
Height (m)	1.69 ± 0.09	1.71 ± 0.09
Weight (kg)	75.58 ± 14.47	78.8 ± 14.63
Gender	6 Male, 2 Female	18 Male, 12 Female
Affected side	5 Right, 3 Left	18 Right, 12 Left
Time Since Injury (months)	1.88 ± 1.73	37.3 ± 34.82
Session time (min)	99.88 ± 15.58	101.36 ± 17.58
Walk Time (min)	71.81 ± 12.05	66.12 ± 17.27
FLACC-Baseline	0.2 5± 0.46	0 ± 0
FLACC-Follow-up	0 ± 0	0 ± 0
FMLE	17.38 ± 7.65	21.97 ± 5.40

*Time Since Injury—Calculated as the number of calendar days from the date of injury to the consent.*

*Fugl–Meyer Lower Extremity Scale (FMLE)—A 34-point motor domain scale that measures the movement, coordination, and reflex action about the hip, knee, and ankle.*

*FLACC Score—The Face, Legs, Activity, Cry. Consolability (FLACC) is a behavioral pain assessment scale used for self-report of pain*.

### Robotic Exoskeleton Device

**Figure 2:**
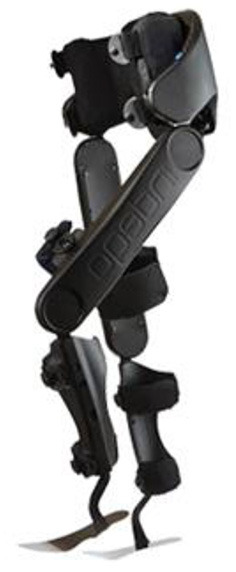
Robotic exoskeleton (Indego) used for over ground walking (http://www.indego.com/).

The Indego (Parker Hannifin, Macedonia, Ohio, USA; Figure 2) is an FDA approved powered lower-limb robotic exoskeleton (RE) for individuals diagnosed with stroke for overground walking. The RE has two active DOF (provided by two motors and embedded sensors and controllers) in the sagittal plane at the hip and knee bilaterally. The device consists of five modular components: a hip segment, a right and left upper leg segment, and a right and left lower leg segment and has a total weight of 26 pounds. The hip component contains a rechargeable lithium ion battery which provides power to the system. At the foot shoe interface there is a built-in carbon fiber ankle foot orthosis which provides some dynamic foot lift and transmission of the weight of the RE to the ground. The RE also provides feedback about the individual's posture and tilt. When the individual transitions from standing to ambulation they move their center of pressure (COP) in the anterior direction to initiate the first step for the walking movement (Tefertiller et al., 2018).

Physical therapists used Therapy+ (Parker Hannifin, Macedonia, Ohio, USA) to control the RE during all overground walking sessions through an Apple iPod touch via Bluetooth connection. Therapy+ software is designed to provide gait training to patients with lower extremity weakness post stroke. The software permits the physical therapist to adjust the level of assistance to each of the participant's joints individually to provide complete or partial assistance as needed. The assistance can be customized to be provided only to the affected limb with the unaffected limb able to move as desired. Participants are required to initiate leg movements to trigger the RE to provide powered assistance at the hip and knee bilaterally. Therapy+ software monitors the patient's behavior and provides auditory and real-time feedback.

### Experimental Procedures

Data are presented for four sessions of overground walking (utilization) in the RE over the course of 2 weeks. Participants completed an initial evaluation and RE fitting before the start of overground walking sessions. Gait speed (meters/second) during the 10 Meter Walk Test (10MWT), gait deviations, and the Functional Ambulation Category (FAC) (Table 2) data were collected before (baseline) and after (follow-up) the four RE walking sessions.

**Table 2:** Primary outcome measures (*n* = 38).

**Outcome measure**	**Description**
10 Meter Walk Test (10MWT)	Participants performed two 10-m walks at a fast, safe walking speed. The time to walk 10 m is recorded using a stopwatch, and 2 m at the beginning and at the end of walk was provided to ramp up and ramp down speed, respectively (total length 14 m). The walking speed (m/sec) was computed as the distance with respect to time to complete the 10 m. The trial with the maximum speed was used for analysis. The average speed was calculated as the sum of speed across all subjects/number of subjects (Kay and Huijbregts, 2003).
Functional Ambulation Category (FAC)	A 6-point scale assesses ambulation status by determining how much supervision and physical assistance the participant required when walking, regardless of whether or not they use an assistive device. The average FAC was calculated as the sum of FAC across all subjects/number of subjects (Holden et al., 1984, 1986).
Gait deviations	Gait deviations were recorded by the physical therapist. The average number of gait deviations was calculated as the sum of gait deviations across all subjects/number of subjects (Perry and Burnfield, 2010).

RE Walking Sessions: All overground walking sessions were administered by a trained therapist using the RE in the partial assistance mode using Therapy+. Participants received training from the physical therapist on the appropriate use of the system. Participants were instructed to walk overground in the RE for up to 30 min per session, they were allowed to take rests or breaks in between the session. The physical therapist adjusted the robotic assistance by changing the joint torques for the hip and the knee in the sagittal plane for the swing and stance phases. The treating physical therapist modified assistance independently for each limb to account for asymmetrical walking function or assist with weakness (post-stroke hemiplegia). Each step of the robot was triggered by patient movement. The RE walking sessions were conducted in a typical gait training environment in the eight clinical centers which included long walkways similar to the conventional overground gait training environments in these clinical centers. Only one walking session in the RE was allowed per day and visits were scheduled in collaboration with adjacent therapies to avoid fatigue. Concurrent therapies were allowed throughout the duration of the study as long as adequate rest was provided for the participant. Outcome measures were taken during the first session (baseline), prior to four sessions of walking in the RE, and during the sixth session (follow-up), after walking in the RE. Selected outcomes measures were collected while participants walked without the RE, no mobility measures were collected while walking in the RE. Vital signs and skin integrity was evaluated by a licensed physical therapist at the beginning and end of each session.

Gait deviations were recorded during a 5-min overground walk without the RE at baseline and follow-up. Gait deviations were observed by a physical therapist using the Rancho Los Amigos Observational Gait Analysis Scale (Perry and Burnfield, 2010). The presence of deviations at the trunk, pelvis, hip, knee, and ankle were recorded (without any indication of severity) throughout the walking trials. The sum of these deviations during all phases of the gait cycle per subject was calculated. The FAC was measured by a physical therapist and categorizes patients based on basic motor skills necessary for functional ambulation. Patients were rated at their most independent level (supervision or physical assistance required to ambulate).

A subset of participants (*n* = 8; four male and four female; six right affected and two left affected, three acute and five chronic; mean ± std. deviation age: 62.4 ± 12.08 yrs.; weight: 64.81 ± 13.28 kg; height: 1.66 ± 0.06 m; TSI: 15.13 ± 25.24 months) from one site walked overground on an instrumented walkway (ProtoKinetics, Havertown, Pennsylvania, USA) without the RE at baseline and follow-up. Participants completed three to six walks and temporal, spatial and symmetry (Table 3) data were used for further analysis to provide preliminary data on changes in gait mechanisms after walking overground with an RE post stroke.

**Table 3:** Secondary outcome measures on a subset of participants (*n* = 8).

**Outcome measure**	**Description**
Step length	The average step length for each gait cycle was computed as the forward linear displacement between foot contact of the ipsilateral leg to foot contact of the contralateral leg during each gait cycle.
Temporal measures	Total time was computed as the time between foot contact of one leg to the subsequent foot contact of the same leg. The average total time was computed for each gait cycle. Further, average swing time for each subject during each condition was computed as the time between the foot off the floor of one leg to foot contact of the same leg during the gait cycle. The stance time was computed as time between foot contact of one leg to foot off of the same leg.
Temporal-spatial symmetry (Holden et al., 1984)	The swing and stance time of each foot during each gait cycle was computed, and the following ratios were used to compute the temporal asymmetry: (1) $$\begin{array}{r} Temporal\;swing\;stance\;symmetry=\left(swing\;time\right)/\left(stance\;time\right) \end{array}$$ Equation (2) was computed for both affected and unaffected limbs (2) $$\begin{array}{r} Overall\;temporal\;symmetry=\left(\frac{affected\;swing\;stance\;symmetry}{unaffected\;swing\;stance\;symmetry}\right) \end{array}$$ The step length of each foot was computed for each gait cycle, and the spatial symmetry ratio was calculated as follows: (3) $$\begin{array}{r} Spatial\;symmetry=\left(\frac{unaffected\;step\;length\;}{affected\;step\;length}\right) \end{array}$$

### Data Analysis

Ambulation metrics are presented from baseline and follow-up assessments (Tables 2, 3).

The one sample Kolmogorov-Smirnov Test (*p* < 0.05) of normality showed that the data was not normal for the 10MWT, FAC, gait deviations- chronic, swing time- unaffected, and temporal symmetry. As a result, the Wilcoxon Signed Ranks test was used to evaluate the effect of four sessions of walking overground in the RE between baseline and follow-up for 10MWT, FAC, gait deviations- chronic, swing time- unaffected, and temporal symmetry.

The one sample Kolmogorov-Smirnov Test (*p* > 0.05) of normality showed that the data was normal for gait deviations- acute, step length, total time, swing time- affected, stance time, and spatial symmetry. As a result, a paired *t*-test was used to determine the effect of 4 sessions of walking overground in the RE between baseline and follow-up for step length, total time, swing time- affected, stance time, and spatial symmetry.

### RE Utilization Data

The session time, walk time, ratio of walk time to up time and number of steps in the device was collected during each RE utilization session from the RE software. Session 1 and session 4 walk time, number of steps, session time, and ratio of walk time to up time was compared. Shapiro-Wilk test of normality (*p* < 0.05) showed that the data was normal for walk time, ratio of walk time to up time and number of steps while session time was not normal for participants in both the chronic and acute stages of recovery. As a result, the paired samples *t*-test was used to evaluate the effect of sessions on walk time, ratio of walk time to up time and number of steps for participants in both the chronic and acute stages of recovery. Session time was evaluated using Wilcoxson signed rank test for participants in both the chronic and acute stages of recovery.

## Results

### Functional Assessments

**Figure 3:**
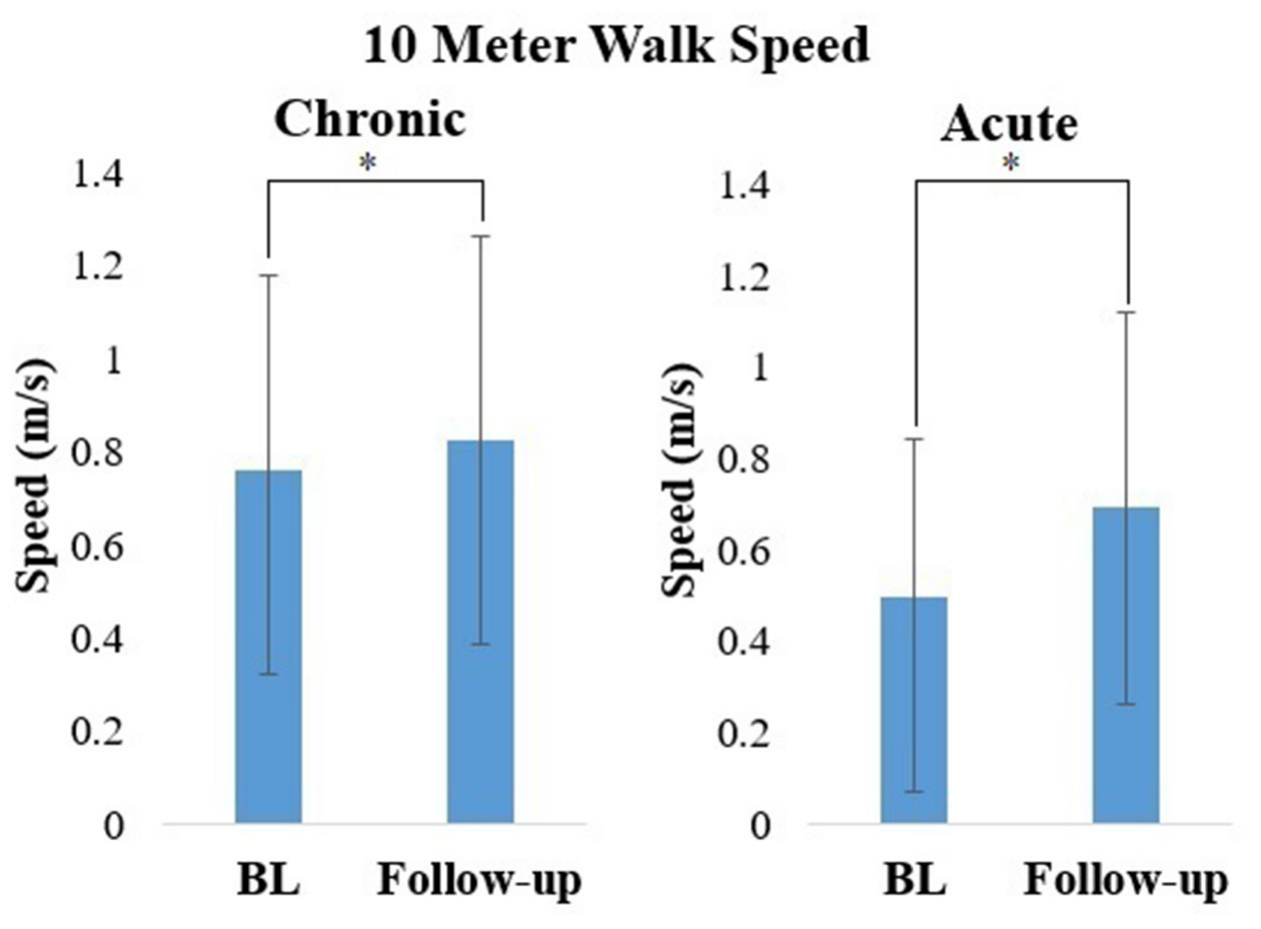
Mean ± standard deviation of Walking speed calculated from the 10MWT during baseline (BL) and follow-up for participants in the chronic and acute stage of stroke recovery. *p < 0.05.

All 38 participants were able to complete four overground walking sessions with the RE. Walking speed significantly increased between baseline and follow-up for participants in the chronic (*Z* = −2.74, *p* < 0.01; Baseline: 0.16–1.54 m/s; Follow-up: 0.26–1.59 m/s) and acute (*Z* = −2.38, *p* < 0.05; Baseline: 0.13–1.09 m/s; Follow-up: 0.24–1.16 m/s) stage of stroke recovery (Figure 3). This suggests that after four sessions of walking overground in the RE, participants increased their walking speed.

**Figure 4:**
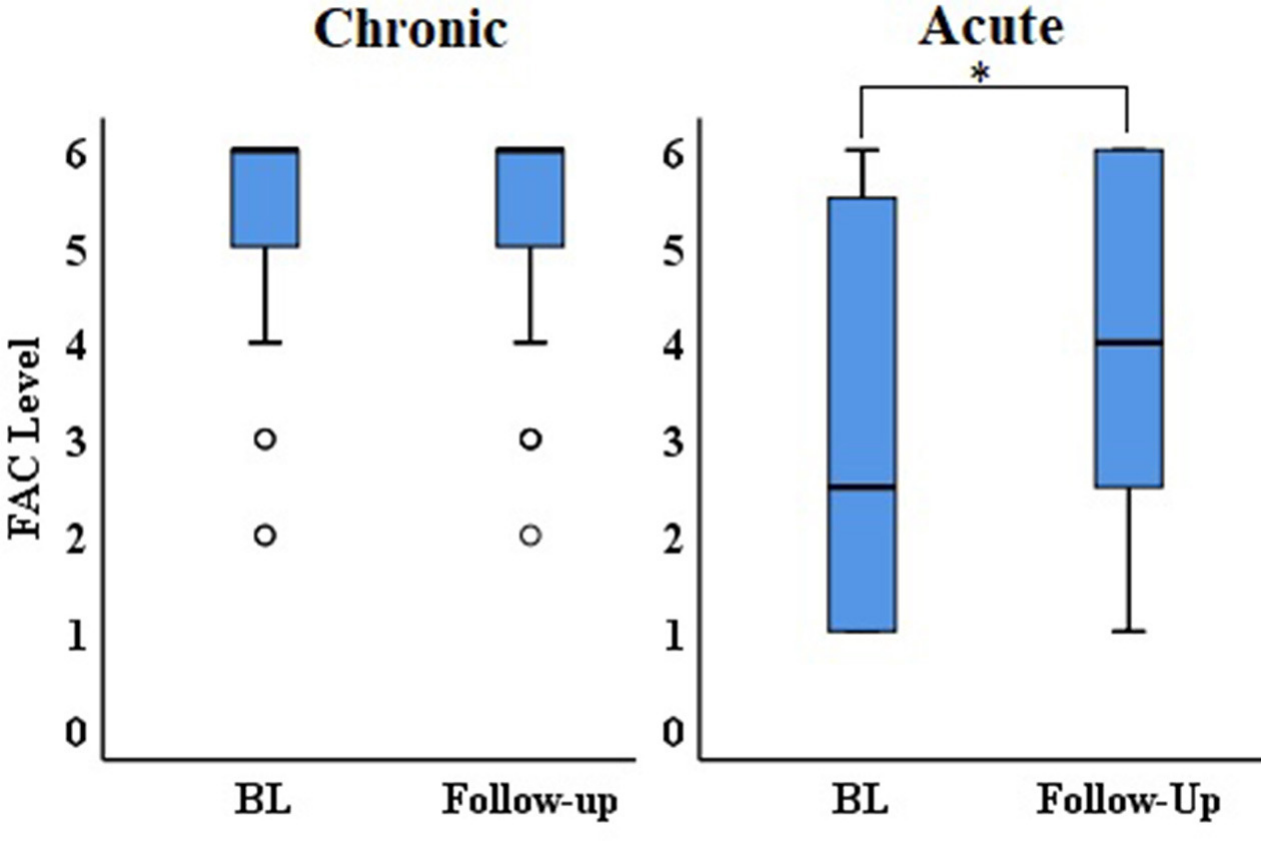
Median and IQR of FAC during baseline (BL) and follow-up for participants in the chronic and acute stage of stroke recovery. *p < 0.05.

FAC level for participants in the acute stages of stroke recovery (*Z* = −2.0, *p* < 0.05) significantly improved between baseline and follow-up (Figure 4). There was no significant change in FAC level between baseline and follow-up for participants in the chronic stage of stroke recovery (*Z* = −0.577, *p* > 0.05).

**Figure 5:**
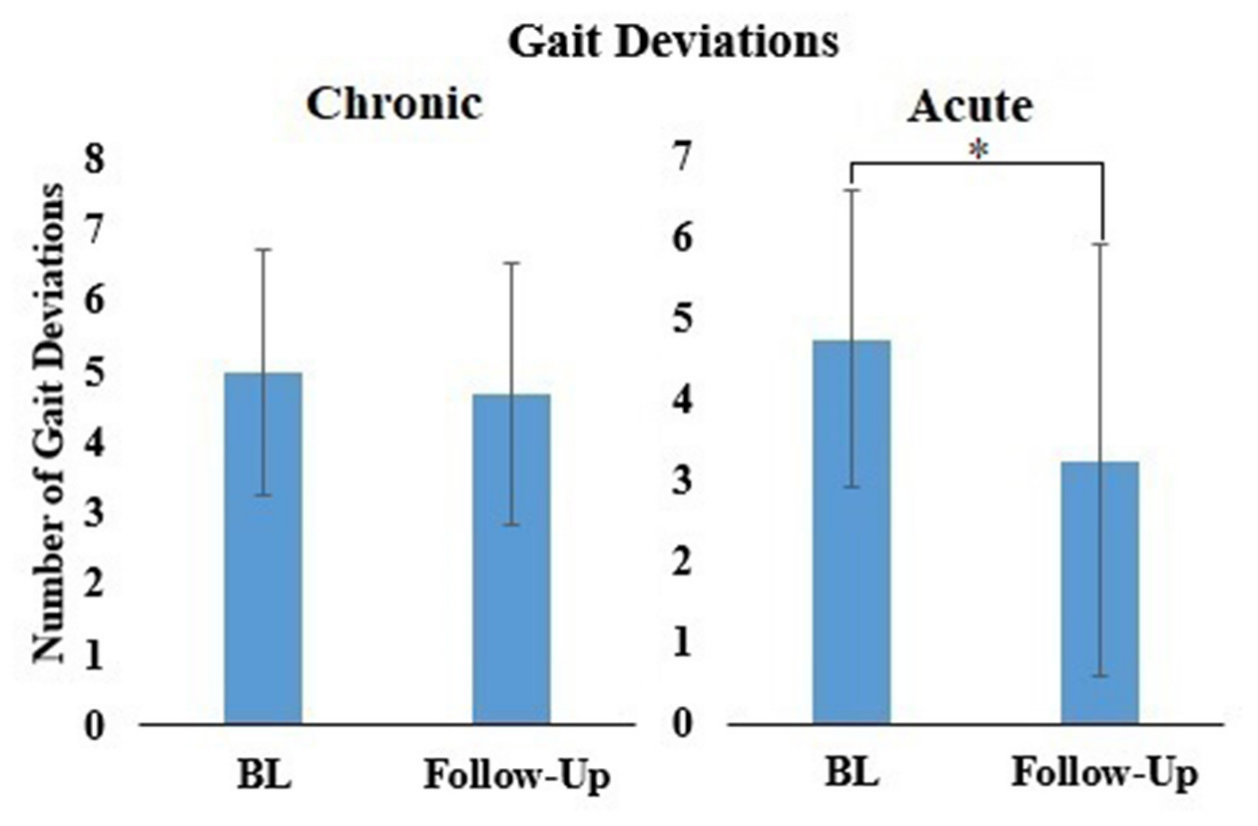
Mean ± standard deviation of Gait deviations for all participants during baseline (BL) and follow-up for participants in the chronic and acute stage of stroke recovery. *p < 0.05.

There were significantly fewer gait deviations observed at follow-up, as compared to baseline for participants in the acute (*t* = 2.646, *p* < 0.05) stage of recovery (Figure 5). This suggests that after 4 sessions of walking overground in the RE, participants walked with fewer gait deviations. There was no significant change in gait deviations level between baseline and follow-up for participants in the chronic stage of stroke recovery (*Z* = −0.729, *p* > 0.05).

### Secondary Analysis Temporal and Spatial Characteristics and Symmetry

A subset of participants walked overground on an instrumented walkway without the RE at baseline and follow-up, outcome variables are presented in Table 4. Swing time significantly increased on the unaffected side (*Z* = −2.521, *p* < 0.05) from baseline to follow-up (Table 4). Total time (affected: *t* = 0.073, *p* > 0.05; unaffected: *t* = −0.069, *p* > 0.05), swing time (affected: *t* = 1.272, *p* > 0.05), and stance time (affected: *t* = −0.119, *p* > 0.05; unaffected: *t* = 0.304, *p* > 0.05) did not show a significant difference from baseline to follow-up.

**Table 4:** Temporal and spatial characteristics (*n* = 8).

	**Metric**	**Baseline**	**Follow-up**
Affected	Total time (s)	2.13 ± 0.90	2.12 ± 0.92
	Stance time (s)	1.50 ± 0.77	1.51 ± 0.81
	Swing time (s)	0.62 ± 0.21	0.59 ± 0.20
	Step length (cm)	38.69 ± 15.86	40.95 ± 14.66
Unaffected	Total time (s)	2.11 ± 0.89	2.12 ± 0.92
	Stance time (s)	1.74 ± 0.94	1.71 ± 0.92
	Swing time (s)	0.37 ± 0.09	0.41 ± 0.11^*^
	Step length (cm)	32.61 ± 18.82	37.99 ± 14.51

* *p < 0.05*.

Step length increased from baseline to follow-up in both the affected (affected: *t* = −0.895, *p* > 0.05) and unaffected (*t* = 1.922, *p* > 0.05) sides (Table 4), but this result was not statistically significant. Overall the change in step length after 4 sessions of overground walking in the RE was higher on the unaffected side compared to the affected side.

**Figure 6:**
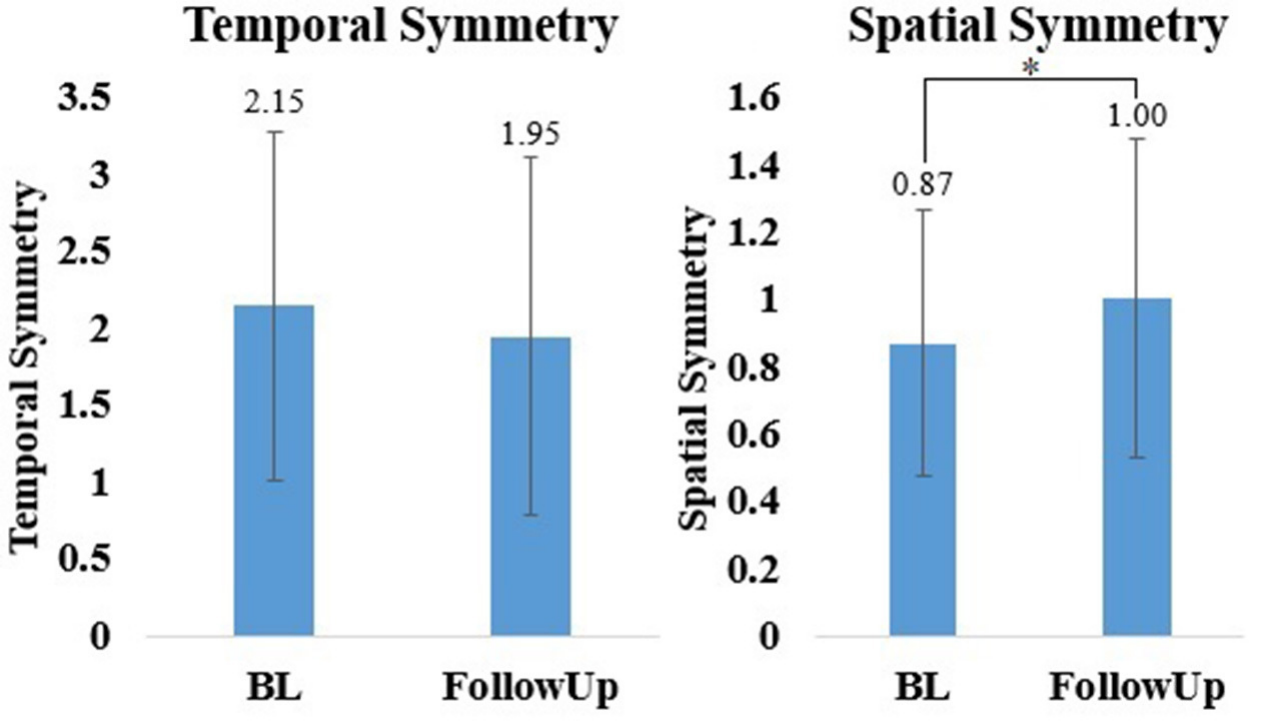
Mean ± standard deviation of temporal spatial symmetry for all participants during baseline (BL) and follow-up. *p < 0.05.

There was significant increase in spatial symmetry (*t* = −2.81, *p* < 0.05) from baseline to follow-up (Figure 6). This suggests that after 4 sessions of overground walking with the RE, the participants improved their spatial symmetry. There was no significant difference in temporal symmetry (*Z* = −1.26, *p* > 0.05) from baseline and follow-up (Figure 6), though a small improvement was observed.

### RE Utilization Data

RE utilization data is shown in Table 5. Number of steps, ratio between walk time to up time and walk time significantly increased from session 1 to session 4 (*p* < 0.05). Session time did not show a significant difference (*p* > 0.05) for participants in the chronic stages of recovery. Number of steps, walk time, session time and ratio between walk time to up time did not show a significant difference (*p* > 0.05) for participants in the acute stages of recovery.

**Table 5:** RE utilization data.

	**Outcome**	**RE session 1**	**RE session 2**	**RE session 3**	**RE session 4**
Chronic (*n* = 30)	Session time (min)	28.6 ± 5.5	27.1 ± 3.7	27.9 ± 5.0	28.2 ± 3.1
	Walk time (min)	14.3 ± 5.4	16.5 ± 5.5	17.4 ± 4.9	18.0 ± 5.6
	Steps	621.2 ± 358.4	726.8 ± 377.8	789.0 ± 372.9	864.1 ± 426.7
	Walk time/Up time (min)	0.5 ± 0.2	0.6 ± 0.2	0.6 ± 0.2	0.6 ± 0.2
Acute (*n* = 8)	Session time (min)	29.2 ± 4.0	24.7 ± 8.1	28.0 ± 2.9	26.2 ± 4.6
	Walk time (min)	12.2 ± 7.6	11.9 ± 8.1	12.7 ± 8.4	13.6 ± 9.9
	Steps	525.6 ± 444.0	513.9 ± 446.6	595.5 ± 518.9	711 ± 588.5
	Walk time/Up time (min)	0.4 ± 0.2	0.4 ± 0.3	0.4 ± 0.3	0.5 ± 0.3

## Discussion

Stroke causes gait and balance deficits affecting mobility and resulting in reduced functional ambulation in the community. The current investigation is focused on presenting preliminary utilization data after walking overground with a robotic exoskeleton (RE) in individuals with acute and chronic stroke. Participants in the acute and chronic stages of recovery had an average contact time of 99 min and 101 min and walked an average of 71 min and 66 min, respectively in the RE (Table 1). Individuals with acute and chronic stroke were provided the same amount of walk time regardless of the impairment level and stage of recovery. The walk time, step count and ratio of walk time to up time significantly increased from first session to the last session in the chronic group (Table 5). Similar trend was also observed in the acute group with number of steps increasing at follow-up. Session time did not increase in both groups, this suggests that participants with varying levels of stroke severity improved their number of steps between sessions with no change in duration of session time in the RE. Participants had varied levels of impairment post stroke ranging from mild to very severe and all tolerated the RE overground walking with no serious adverse events or pain (FLACC) reported during the study. There were no study-related serious adverse events or falls reported across the eight rehabilitation centers due to the RE overground walking. The participants also continued their standard of care if they had any ongoing therapy at the time of study enrollment. The results demonstrated an increase in walking speed, functional ambulation category, and a decrease in gait deviations from baseline to follow-up for participants post stroke in the acute and chronic stages of recovery. In addition, spatial symmetry and step length also improved in the subset of the participants.

Gait speed is a commonly used metric to assess the functional capacity of people with stroke to ambulate within the household or community. According to Perry et al. individuals with a walking speed between 0.4 and 0.8 m/s would be considered limited community ambulators (Perry et al., 1995). Therefore, an increase in gait speed could potentially result in improved functional mobility. In this preliminary evaluation, gait speed increased from baseline to follow-up in both the acute and chronic stages of recovery. Participants in the acute stages of recovery showed an increase from limited household ambulators and approaching community ambulators. The minimally clinically important difference (MCID) scores for walking speed post stroke range from a small meaningful change of 0.05 m/s and a large meaningful change score of 0.10–0.16 m/s (Perera et al., 2006; Tilson et al., 2010). The MCID is a commonly used criteria to evaluate the clinical significance of an intervention. After 4 sessions of walking overground in the RE individuals increased their walking speed an average of 0.07 m/s in the chronic group and 0.19 in the acute group, both meeting the MCID criteria for a meaningful change in walking speed.

FAC categorizes patients based on basic motor skills necessary for functional ambulation. At baseline, participants in the acute group required manual contact of at least one person for balance, and coordination during overground walking. At follow-up, the participants were progressing toward ambulation with supervision according to the FAC scale (closer to four) in the acute group. Scoring ≥4 on the FAC following an inpatient rehabilitation program is predictive of community ambulation at 6 months (Mehrholz et al., 2007). Hence, RE overground walking could have contributed to improved FAC in the acute group. FAC level of participants in the chronic group was six at baseline (the highest score in FAC) and participants were ambulating independently without supervision on all surfaces.

Observational gait deviations were recorded by the physical therapists during overground walking at a self-selected pace. The number of gait deviations decreased in both groups from baseline to follow up, where the number of gait deviations in the acute group showed a significant change. Reduced post stroke gait deviations could signify a decrease in pathological compensations for ambulation. Typical post stroke treatment includes strengthening or learned compensation for weak muscles, which can reduce compensatory gait deviations (Perry and Burnfield, 2010). The results suggest that there may have been a therapeutic benefit of walking overground in the RE.

Participants who walked overground in the instrumented walkway provided preliminary information on mechanistic changes in gait after walking in the RE which might have contributed to the improvements in functional ambulation. Spatial symmetry significantly improved after walking overground in the RE. Additional improvements were observed in temporal symmetry. Inter-limb symmetry is the ability to maintain temporal and spatial symmetry between the limbs, which results in a healthier gait pattern with less gait deviations. Asymmetrical stepping is a characteristic of hemiparetic walking and a result of sensorimotor deficits post-stroke. Temporal and spatial symmetry outcomes (i.e., performance paretic leg with respect to non-paretic leg) can provide insights into underlying impairments (Kinatinkara Balasubramanian, 2008). After walking in the RE temporal and spatial symmetry improved, indicating that the bilateral sagittal plane assistance provided by the RE during walking may have a therapeutic effect on gait symmetry. Symmetry closer to one signifies that both limbs are performing a symmetrical movement (Patterson et al., 2008). Previous research has shown that there is a correlation between deviations in temporal and spatial symmetry and balance (An et al., 2017) and reduced walking speed (Balasubramanian et al., 2007; Wonsetler and Bowden, 2017). The observed decrease in gait deviations, improved gait mechanics and balance could have a direct effect on gait speed, and FAC (Li et al., 2018).

The results show the ability of RE utilization with acute and chronic stroke with varying degrees of severity. Though the preliminary results are promising a potential limitation in this investigation is the small sample and presence of concurrent standard of care therapies. The results especially in the acute stages of recovery might have also been influenced by the spontaneous plasticity. Hence, Future studies with a more controlled training environment (duration and intensity of training) and comparison to standard of care (control group) is required to further validate the efficacy of RE overground gait training in acute and chronic stroke.

## Data Availability Statement

The datasets presented in this article are not readily available because current Institutional Review Board approvals does not include data sharing with external institutions. Requests to access the datasets should be directed to Karen J. Nolan, knolan@kesslerfoundation.org.

## Ethics Statement

The studies involving human participants were reviewed and approved by Kessler Foundation, Cedars-Sinai Medical Center, Craig Hospital, Sheltering Arms Physical Rehabilitation Centers, St. Charles Hospital, Shirley Ryan AbilityLab, University of Texas at Houston McGovern Medical School, and Shepherd Center. The patients/participants provided their written informed consent to participate in this study.

## Author Contributions

KN, GF, and AJ designed the study and collected the data. KK and KN analyzed the data and drafted and finalized the manuscript. GF edited and finalized the manuscript. CT, DL, JZ, PR, and AW collected the data. All authors contributed to the manuscript.

## Conflict of Interest

The authors declare that the research was conducted in the absence of any commercial or financial relationships that could be construed as a potential conflict of interest.

## Publisher's Note

All claims expressed in this article are solely those of the authors and do not necessarily represent those of their affiliated organizations, or those of the publisher, the editors and the reviewers. Any product that may be evaluated in this article, or claim that may be made by its manufacturer, is not guaranteed or endorsed by the publisher.
